# Beyond the Protocol: Revisiting the Critical Role of Donor Plants in Cryopreservation of Economically Important Clonal Crops

**DOI:** 10.3390/plants15081221

**Published:** 2026-04-16

**Authors:** Elena Popova, Haeng-Hoon Kim

**Affiliations:** 1 K.A. Timiryazev Institute of Plant Physiology, Russian Academy of Sciences, Botanicheskaya 35, Moscow 127276, Russia; 2 Department of Agricultural Life Science, Sunchon National University, Suncheon 57922, Republic of Korea

**Keywords:** cryobanking, cryoprotectant toxicity, cold acclimation, donor plants, droplet-vitrification, encapsulation, shoot tips, sucrose pretreatment, stress tolerance, vitrification

## Abstract

Shoot tip cryopreservation is essential for the long-term conservation of plant genetic resources. It provides the only reliable method for establishing a long-term, readily available gene pool of clonally propagated crops and elite in vitro clones used in the pharmaceutical, food, and cosmetic industries. Still, its success is often limited by the inherent sensitivity of many species to the osmotic and chemical stresses imposed by concentrated cryoprotectant (vitrification) solutions and severe dehydration. The optimization of modern cryopreservation protocols primarily focuses on modifying shoot tip preculture, cryoprotectant treatments, or regrowth conditions, while frequently overlooking donor plant preconditioning or relegating it to a secondary role. However, the physiological state of in vitro plants from which apical or axillary shoot tips are extracted may hold the key to successful post-cryopreservation recovery, especially in cryo-sensitive taxa. This review revisits the critical role of donor plant vigor and induced stress tolerance in the cryopreservation of clonal crops by systematically evaluating preconditioning strategies, including cold acclimation, sucrose pretreatment, and the use of growth regulators and signaling molecules such as abscisic, jasmonic, and salicylic acids, involved in stress signaling and tolerance development. The beneficial physiological changes induced by donor plant pretreatment, such as reduced freezable water content and the accumulation of protective compounds, are discussed in the context of contemporary cryopreservation methods. The effects of culture conditions, including the roles of ammonium and nitrates, light quality, culture density and aeration, medium strength, culture age, and subculture duration, are also considered. We analyze how different treatments of in vitro donor plants improve shoot tip tolerance to osmotic and/or chemical toxicity imposed by specific cryopreservation methods to support a material-centered selection of a cryopreservation procedure. Future directions and potential approaches for integrating target donor plant preconditioning into modern cryopreservation protocols for shoot tips, particularly in stress-sensitive species, are discussed.

## 1. Introduction

### 1.1. Role of Cryopreservation in Biobanking Plant Genetic Resources

**Figure 1:**
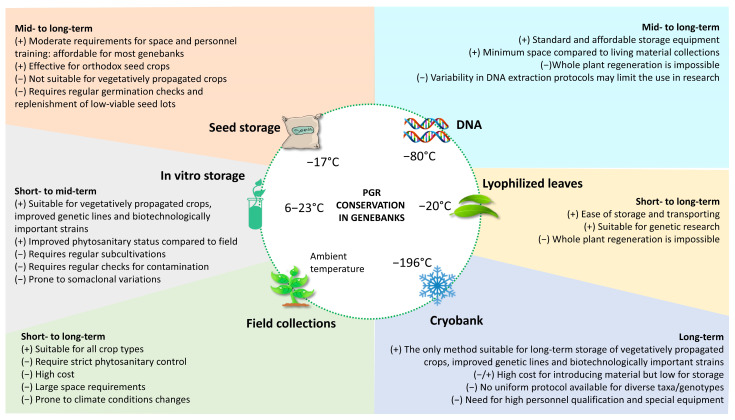
Diversity of methods for conserving plant genetic resources (PGRs) in genebanks, their feasibility for different types of plant germplasm, and major limitations.

Cryopreservation, the long-term storage of biological materials at cryogenic temperatures, predominantly in liquid nitrogen (−196 °C, LN), is currently one of the fundamental strategies for ex situ conservation of plant genetic resources (PGRs) in genebanks [1] (Figure 1). This strategy is crucial for the secure preservation of plants producing non-orthodox seeds, vegetatively propagated cultivars, genetically improved lines, and plant biotechnology products (cell lines, embryogenic tissues, hairy- and adventitious roots, etc.), ensuring their availability for future generations [2,3,4,5]. In plant genebanks, active collections, such as field-grown plants or in vitro cultures, are readily available for evaluation, research, and distribution, while base collections are in long-term storage (LTS) and usually serve as a secure backup in the event of the loss of active collections [6]. Although cryopreservation is the newest addition to the germplasm storage system, it is acknowledged as the most reliable option for LTS because it provides the highest level of protection for important traits and the genetic integrity of the germplasm over the long term [5].

Cryopreservation has evolved from the 19th-century seldom experiments with seeds frozen in liquefied gases [7] to a routine technique used worldwide to preserve plant heritage [1,8], but this progress was not smooth. In the history overview collated by Prof. Erica Benson, she emphasized that the development of cryopreservation protocols transcends the disciplinary boundaries of medical, plant, and animal cryobiology and was largely based on the combination of fundamental and applied research [9]. The milestones on this path were set by Maximov, who first described the protective effects of glycerol and sugars in plants exposed to freezing temperatures [10,11], and by Polge et al. [12], who discovered the cryoprotective properties of glycerol applied to fowl spermatozoa. Further studies formulated multiple cryoprotective mixtures based on glycerol, sugars, dimethyl sulfoxide (DMSO), and other compounds to protect plant tissues during the freeze–thaw cycle in cryopreservation [13]. Maximov also postulated that plants were killed at freezing temperatures by the accumulation of ice crystals between cells, which dehydrate and mechanically damage cells, leading to the coagulation of cell solutes (cited by [9]). These early investigations supported the formulation of the two-factor theory of freezing injury proposed by Mazur, Meryman, and their colleagues [14,15]. Later studies by Steponkus et al. (reviewed in [16]) highlighted the plasmalemma’s leading role in the mechanism by which plant cells perceive and survive cold stress. In 1956, Prof. Akira Sakai [17] reported the survival of cold-hardened and prefrozen mulberry twigs following LN exposure, marking the dawn of cryopreservation for vegetative plant parts. These and subsequent studies demonstrated that dehydration is critical for plant material to survive freezing and established vitrification, the physical transformation of water remaining in cells from liquid to a glassy state, as the major mechanism enabling living cells to survive cryogenic exposure. Further investigations provided evidence that the survival of plant tissues after cryopreservation could be correlated with cold hardiness [18,19,20]—an effect widely adopted in cryopreservation methods applied to PGR. Currently, over 10,000 accessions of economically important crops are safely preserved for the long term through cryopreservation [5,8,21]. More than 80% of these belong to the potato, cassava, banana, mulberry, garlic, apple, and mint groups. The lead role is played by the international genebanks such as International Potato Center (CIP), Bioversity International (BI) and International Institute of Tropical Agriculture (IITA) as well as advanced national genebanks such as National Laboratory for Genetic Resources Preservation, US Department of Agriculture (NLGRP USDA), Leibniz Institute of Plant Genetics and Crop Plant Research (IPK) and Julius Kühn-Institute (JKI) Germany, Chinese Academy of Agricultural Sciences (CAAS) China, Tissue Culture and Cryopreservation Unit, National Bureau for Plant Genetic Resources (NBPGR) India, Genetic Resources Center, National Agriculture and Food Research Organization (NARO) Japan, Rural Development Administration (RDA) Republic of Korea, Crop Research Institute (CRI) Czech Republic, and others as collated in the recent reviews [1,8]. However, beyond this encouraging progress, many crops and endangered plant species, particularly from the tropics, remain recalcitrant to cryopreservation. The main issues stem from the inherited sensitivity of these species to dehydration and low temperatures, and from the lack of a uniform (standard) protocol applicable to all taxa—problems that are difficult to overcome using the existing cryopreservation methodology.

### 1.2. Limitations of the Existing Cryopreservation Protocols

Recent reviews [1,3,5,22,23,24,25] provide a comprehensive analysis of cryopreservation methods and their feasibility for cryobanking various types of plant propagules. Among the diverse explants, meristematic tissues such as apical or axillary buds are widely regarded as the preferred material, as they best ensure the preservation of genetically identical samples and the production of true-to-type plants post-preservation [22,26].

**Figure 2:**
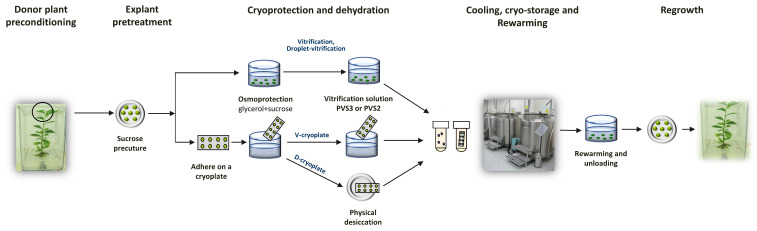
Schematic representation of droplet-vitrification, D- and V-cryoplate methods for cryopreservation of the shoot tips, illustrating the complex, multi-stage layout of these protocols.

Traditional cryopreservation protocols are based on controlled (programmed, or slow) freezing, in which plant material is dehydrated during cooling at a low, constant rate in a mid-concentrated cryoprotectant mixture. In more modern vitrification-based methods, material is dehydrated and cryoprotected in concentrated cryoprotectant (vitrification) solutions before rapid immersion in LN [27]. The vitrification approach was further modified by performing cooling and rewarming steps on aluminum foil strips using drops of vitrification solution (droplet-vitrification, DV), thereby increasing the rate of temperature exchange and reducing the likelihood of ice crystal formation [28]. Another alternative is the encapsulation of material into alginate beads, followed by desiccation under airflow or with silica gel (encapsulation-desiccation), or by vitrification solutions (encapsulation-vitrification) [29,30]. A combination of these methods is the cryoplate technique, in which an alginate-coated material is adhered to aluminum plates and either dehydrated (D-cryoplate) or treated with a vitrification solution (V-cryoplate) [31]. In any of these protocols, a sequence of steps is required (Figure 2), including selection and preparation of donor material, a series of pretreatments for dehydration and/or cryoprotection to ensure vitrification of plant tissues upon LN exposure, cooling and rewarming, and ultimately, the regeneration of viable plants [27]. The integrity of this entire chain is critical, as suboptimal conditions at any stage can lead to complete failure or reduced viability [23].

The principal limitation of these methods is the significant cytotoxicity of the concentrated cryoprotective agents (CPAs), which must be applied for extended periods [23]. CPAs are essential for replacing water, lowering freezing points, and enabling vitrification (the transition of water into a glassy state), thus preventing damaging ice crystal formation inside cells during cryopreservation [32,33]. However, the concentrated CPA used in modern cryopreservation protocols imposes severe osmotic and chemical stresses on living cells [33,34]. To mitigate the toxic effects of CPA, a range of strategies were developed [13,35,36,37]. Extensive research led to the formulation of CPA mixtures with a balanced ratio of permeating and non-permeating CPAs, ensuring rapid dehydration and cryoprotection of organized tissues [13,33]. The most commonly used plant vitrification solutions, PVS2 (containing 30% glycerol, 15% dimethyl sulfoxide (DMSO), 15% ethylene glycol (EG), and 13.7% sucrose, [38]) and PVS3 (50% glycerol and 50% sucrose, [39]), remain standard for decades [22,27]. While often effective, they are not universal. Although alternative CPA formulations were developed and implemented with varying degrees of success [13,33,36,40], none achieved widespread adoption of PVS2 and PVS3.

The tolerance of each plant material to the toxic effects of CPAs during cryopreservation is often assessed through trial and error. Explants are exposed to CPAs for varying durations to determine the optimal time frame at which the protective and toxic effects of CPAs are most balanced. Reducing CPA exposure temperatures and enhancing explant tolerance through a series of pretreatments with osmolytes (sucrose, sorbitol) or proline are also effective. Other studies recommended adding antioxidants (e.g., ascorbic acid, polyvinylpyrrolidone [41,42,43], as well as multifunctional molecules such as melatonin [44,45,46] during the cryopreservation procedure [25]. In addition, post-rewarming conditions were modified by adjusting the cryoprotectant removal solution (unloading step) [47], the composition of growth regulators in the recovery medium [48,49,50], and the quality or intensity of light [51], and these modifications were intensively investigated [25,52,53].

Consequently, most current research on plant cryopreservation focuses on optimizing preculture conditions, CPA treatment (composition, exposure duration and temperature), and regrowth conditions. In contrast, the critical importance of the donor plant’s physiological state and quality, i.e., the very first stage of the whole procedure, is often overlooked.

This review highlights the important role of donor plant vigor in modern cryopreservation protocols. We also explored whether literature analysis may reveal the advantage of donor plant preconditioning in specific cryopreservation methods, or whether it correlates with shoot tip sensitivity to osmotic or chemical stresses during cryopreservation.

## 2. A Systematic Approach to Cryopreservation: Osmotic vs. Chemical Tolerance

To overcome limitations of empirical, trial-and-error optimization of the cryopreservation protocol, a systematic approach was proposed, centered on the droplet-vitrification (DV) method [40]. Instead of researching for a universal protocol, it provides a structured framework for identifying the tolerance/sensitivity of plant material to chemical and osmotic stresses provoked by PVS [54]. Beyond standard PVS2/PVS3, the systematic approach incorporates alternative vitrification solutions (e.g., VS A3, VS B5) and osmoprotection solutions (e.g., C4-35%) [36,55] as listed in Table 1. These alternatives allow for balancing osmotic potential and chemical composition (e.g., higher sucrose/glycerol vs. lower DMSO/EG), providing a “toolkit” to fine-tune the stress imposed on the tissue.

Further, in the systematic approach, rather than random screening, plant materials are classified into four groups based on their size, structure, and, most importantly, their responses (tolerance or sensitivity) to osmotic and chemical stresses induced by CPAs [40]. A specific set of pre-defined protocol variations (typically 10–15 treatments) is then applied based on this classification [40]. The key part of tolerance prediction is comparing regrowth after CPA treatment without LN exposure (CPA toxicity test) with regrowth after the full cryopreservation cycle (CPA + LN) [59]. This approach has been shown to drastically reduce the number of explants and experiments required to achieve satisfactory regrowth after cryopreservation [40,54]. Moreover, it provides a predictive framework and increases the likelihood of successful recovery for new (untested for cryopreservation) plant taxa [49,60,61,62].

It is noteworthy that all CPA solutions induce both chemical and osmotic stress, but the predominant effect varies depending on the components’ osmotic activity and chemical toxicity. Chemical stress is thought to be mainly provoked by DMSO and EG—common components of PVS2, VS A3, and their analogs. In contrast, osmotic stress is mainly associated with PVS3, VS B5, and their alternatives, which contain high concentrations of glycerol and sucrose.

Table 2 illustrates the differential response of plant materials to CPA-induced osmotic and chemical stress, compiled from published cryopreservation protocols. For illustrative purposes, the tolerance level is assessed by measuring the regrowth of shoot tips treated with a vitrification solution without cryopreservation under the same conditions that yield maximum regrowth following LN exposure. As demonstrated in Table 2, several species tolerate relatively long exposure to chemical (PVS2) or osmotic (PVS3) stress, with regrowth above 80%. In contrast, others are susceptible to both, showing regrowth below 60% even in the best treatments without cryopreservation. There are also multiple examples of variable sensitivity among different varieties of the same species, some of which are shown in Supplementary Table S1

## 3. Linking Donor Plant Treatment with Cryopreservation Protocols

Advanced protocols such as DV-, V-, and D-cryoplate methods greatly reduced the dependence of cryopreservation success on donor material quality. They enabled cryobanking of plant taxa that never experience low temperatures in their natural habitats. However, the high sensitivity of some taxa/genotypes to both osmotic and chemical stresses poses a persistent challenge that cannot be fully resolved through protocol modifications. We hypothesized that specific pretreatments of donor plants could be incorporated into these modern protocols to improve regeneration.

Strategies for donor plant preconditioning before cryopreservation are limited and include (i) cold acclimation (CA), (ii) preculture on medium with increased concentrations of osmotically active or stress-related compounds such as sucrose, sorbitol or proline, (iii) addition of antioxidants, (iv) addition of specific growth regulating or signaling molecules, (v) adjustment of medium salt composition by removing or substituting ammonium, (vi) manipulating light, culture density, aeration, and other factors related to cultivation conditions.

In the following sections, we review representative examples of how donor plant preconditioning and physiological state influenced cryopreservation outcomes. We also explored whether specific preconditioning methods can improve tolerance to distinct stresses, such as osmotic or chemical cytotoxicity, or both.

### 3.1. Cold Acclimation of Donor Plants

Cold acclimation (CA), also known as cold hardening, is the preconditioning of donor plant material at low temperatures under a shifted light regime or in darkness to induce physiological changes associated with freezing tolerance [78]. This treatment is intended to mimic the natural process of preparing plants for colder seasons. In particular, alternating day–night temperatures resemble the night frosts typical of temperate climates.

The effects of CA on plants have been comprehensively investigated, as reviewed by [78,79,80]. CA stress leads to activation of multiple signaling pathways related to Cold-Responsive Genes (COR), C-repeat binding factors (CBFs), Ca^2+^ signaling, and Mitogen-Activated Protein Kinases (MAPKs) regulatory networks in plant cells, leading to alteration in gene expression, followed by changes in plant physiology, affecting also the shoot tips [30,80,81,82]. The process triggers the accumulation of soluble sugars and starch, enhances the synthesis of osmoregulatory amino acids, such as proline [83], and of specific proteins, including Late Embryogenesis Abundant (LEA) proteins, Anti-freezing Proteins (AFPs), and Cold Shock Proteins (CSPs) [78]. At the cell membrane, the primary site of cold sensing and low-temperature-induced damage, CA increases phospholipid content, mostly due to diunsaturated species [18,84]. Reactive Oxygen Species (ROS), including superoxide (O2˙−), hydroxyl radicals (OH−), and hydrogen peroxide (H_2_O_2_), are often associated with various stresses in plants, including cold stress. The development of oxidative stress in plant material during and after cryopreservation is considered one of the major factors to reduce regrowth [27,41]. Thus, CA may also induce moderate oxidative stress, thereby activating both enzymatic and non-enzymatic ROS-scavenging systems [85].

In cryopreservation, CA was widely applied to temperate crops that possess an inherent ability to cold-harden. Before conducting a literature review, we hypothesized that the physiological changes induced by CA better prepare plants for controlled (programmed) cryopreservation. This procedure mimics the natural, gradual temperature reduction, accompanied by slow protoplast dehydration due to extracellular ice. CA may also be useful in the vitrification method when it involves prolonged explant treatment with PVS2 or its alternatives at low temperature (usually at 0 °C). Yet, CA may not be the best choice to combine with PVS3 due to the much more severe osmotic stress it imposes.

This hypothesis was confirmed by a literature analysis (Table 3; see also the extended version in the Supplementary materials, Table S2).

CA of donor plants was widely used in the 1980s–2000s to cryopreserve temperate crops such as apple, pear, currant, strawberry, mint, and some forest trees, including birch and walnut, using controlled freezing (31% of all studies analyzed). When combined with the controlled freezing protocol, CA of donor plants improved post-cryopreservation regeneration by 18–26% in *Betula pendula* [86] and 29–57% in *Rubus* spp. [87,88], 48% in *Vaccinium* spp. [89], 33–54% in *Malus* spp. [90], and 63–84% in *Pyrus* spp. [91]. However, even for temperate crops, CA efficacy is highly conditional, varying by crop type and genotype. Examples from *Rubus*, *Vaccinium*, potato, and pear demonstrate that some cultivars respond positively to CA, with significantly improved regrowth, while others show negligible or even negative responses.

Among vitrification-based methods, CA was most frequently combined with PVS2-based protocols (44% of all studies analyzed). Many of them involved prolonged PVS2 exposure at low temperature, e.g., up to 60 min for *Actinidia chinensis* [42], 80 min for *Malus* spp. [90], 120–150 min for *Fragaria* × *ananassa* [92,93], and even 180 min for *Mentha* spp. [94]. When CA of donor plants was combined with DV and vitrification in cryotubes, it notably improved regrowth after cryopreservation in *Actinidia chinensis* and *Mentha* spp. by 30–35% [95,96], in *Malus* spp. (50–68%, [90,97]), and *Juglans regia* (60–68% [98]) among other crops (Table 3). CA of donor plants is particularly useful for *Solanum* spp. allowing for 30–50 min PVS2 exposure at 0 °C and is recommended as a part of the routine protocol for potato shoot tip cryopreservation in CIP (Peru) and IPK (Germany) [99,100,101].

CA induces beneficial biochemical changes, including accumulation of soluble sugars, alterations in membrane lipids, and modifications in protein metabolism, resulting in improved tolerance to osmotic and freezing-induced dehydration and chilling. For example, improved regrowth of cold-acclimated potato shoot tips was associated with higher total soluble sugar concentrations (glucose, fructose, and sucrose) than in control cultures maintained under normal conditions [83]. Glucose levels were particularly elevated after CA, while fructose and sucrose showed only minor changes. Similarly, in the work by Folgado et al. [102], a significant positive correlation was found between sucrose concentration in CA-donor plants and post-cryopreservation shoot tip regrowth. The proteome analysis revealed that proteins involved in carbon metabolism were most affected by CA treatments. Changes in the abundance of certain proteins involved in stress responses and oxidative homeostasis were also reported, including proteinase inhibitors and heat shock proteins [102]. This aligns well with the general perception that carbohydrate metabolism in plants is very sensitive to low-temperature stress [103]. In mint, cryo-scanning electron microscopy (cryo-SEM) revealed that axillary shoot tips excised from cold-acclimated nodal segments and cryopreserved using the same DV protocol had smaller ice crystals, predominantly within the vacuolar space. This finding indicates a higher cytoplasmic solute concentration relative to explants without CA [104].

As shown in Table 3, there were very few cases of CA combination with PVS3-based vitrification protocols (4 out of 32 studies) or with encapsulation-dehydration (5 studies) (Table 3). The PVS3-based studies with CA of donor plants reported cryopreservation of apices or bulbils of *Allium*, *Lilium*, and *Arabidopsis*, i.e., taxa and plant materials that are naturally tolerant to both osmotic and chemical stresses. These results are expected because both PVS3 and dehydration-based methods impose rapid, severe osmotic stress that cold-acclimated plant material cannot fully accommodate.

In summary, CA of donor plants remains effective in combination with controlled freezing and PVS2-based vitrification methods. Moreover, it may be particularly useful for preparing explants for prolonged low-temperature exposure to PVS2. Yet, it is probably insufficient to adapt osmotic-sensitive and medium-sensitive materials to PVS3 or to desiccation-based protocols. Another important limitation of CA is that it applies only to crops capable of cold hardening under low-temperature/light regimes and is generally not suitable for crops from tropical regions. Further, recent studies have demonstrated that for some species, CA may be bypassed by optimizing preculture and cryoprotection conditions, particularly by stepwise preculture of shoot tips with increasing sucrose concentrations at ambient or low temperatures. This phenomenon was demonstrated across several crops, including strawberry [105,106], a range of potato genotypes [107,108], mint [109,110], and lily [111], as well as *Alnus glutinosa* [112], *Actinidia chinensis* [42], *Betula pendula* [113], and *Chrysanthemum morifolium* [29]. In other crops, CA may be effectively replaced by sucrose pretreatment of donor plants, as discussed in Section 3.2.

**Table 3:** Use of cold acclimatization in cryopreservation of shoot tips of various crops. Only representative examples for each crop are included. The extended version of this table is available in Supplementary Materials Table S2.

Species	CA Treatment	Method *	Cryopreservation Procedure **	Survival (S) or Regeneration (R) ***	Reference
*Actinidia chinensis* var. *chinensis* ‘Hort16A’	4 °C for 2 weeks, 10 h photoperiod (25 μmol m^−2^ s^c^ )	DV	0.25 M–0.5 M– 0.75 M–1.0 M sucrose + 0.4 mM ascorbic acid (1 day each, 4 °C) → 2M glycerol + 0.4 M sucrose 20 min 0 °C → PVS2 0 °C 60 min	R: 40% vs. <10% without CA and explant preculture with sucrose	[42]
*Allium*, 6 genotypes	25/−1 °C under 16 h photoperiod (60–80 μmol m^−2^ s^−1^ ) or 2 °C same light for 10–12 weeks → 5 °C 8h light/16 h darkness + 100 µM L^-1^ ABA for 28 days	Vit	0.3 M sucrose 1 day → 0.4 M sucrose + 2 M glycerol for 20 min → PVS3 for 120 min	R: ~46–100% vs. ~6–40% depending on genotype	[114]
*Betula pendula*	NH_4_NO_3_ and Ca(NO)_3_ were substituted by KNO_3_ (10 mmol L^-1^)	CF	0.5% DMSO + 100 µM L^-1^ ABA for 72 h (5 °C 8/16 h light/dark) → 10% PEG + 10% glucose + 10% DMSO 30 min + 30 min at 0 °C	R: average 58.3% (15–88.3% for 4 genotypes, genotype-dependent response)	[113]
*Chrysanthemum morifolium* cv. shuhounochikara	10 °C 18 h light/16 h darkness for 3 weeks	Enc-deh	0.3 M sucrose at 5 °C in darkness for 3 days → 2 M glycerol + 0.4 M sucrose in beads 1 h	R: 85%	[29]
		Vit	0.3 M sucrose at 5 °C in darkness for 3 days → 2 M glycerol + 0.4 M sucrose 20 min → PVS2 20 min	R: 85%	
*Dioscorea bulbifera*, *D. polystachya*, *D. cayenensis*, *D. alata*	28 °C/5 °C 12 h light/12 h darkness for 3 weeks	DV	10 or 15% sucrose 3 days → 13.7% sucrose + 18.4% glycerol 20 min → PVS2 20 min	R: 30–47% 3 species; 0% for *D. alata*	[115]
*Fragaria* spp. Method tested for 107 cultivars and 20 wild species (51 accessions)	22 °C 8 h light/−1 °C 16 h darkness	Vit	5% DMSO and 0.85% agar for 2 days under CA conditions → 2.0 M glycerol + 0.5 M sucrose for 15 min → PVS2 for 2.5 h at 0 °C	R: average 89.55% for cultivated accessions and 85.5% for wild accessions	[92]
*Juglans regia*, 4 genotypes	22 °C/−1 °C 8 h light/16 h darkness for 5 weeks, 10 μmol m^−2^ s^−1^	Vit	0.3 M sucrose for 2 days → 2 M glycerol + 0.4 M sucrose for 20 min → PVS2 80 min at 0 °C	R: 59.9–67.8% vs. ~0% without CA	[98]
*Juncus effusus* line ‘NZ219’	5 °C 8 h light/16 h darkness for 30 days	Vit	0.3 M sucrose at 5 °C for 3 days → 2 M glycerol + 0.4 M sucrose 30 min → PVS2 for 40 min	R: ~70% after 30–60 days of CA vs. ~23% without CA	[116]
*Lilium* spp. Method applied to 160 accessions	4 °C 16 h light/8 h darkness, 35 μmol m^−2^ s^−1^ for 7 days	DV	0.3 M sucrose 24 h → 0.7 M sucrose 17–24 h → 35% PVS3 for 40–60 min → PVS3 for 90–240 min	R: 54.3–58.5% for 160 accessions	[72,117]
*Malus domestica* (4 cultivars), *M. sieversii* (1 cultivar)	22 °C/−1 °C 8 h light/16 h darkness for 3 weeks	Vit	0.3 M sucrose for 2 days under CA conditions → PVS2 80 min 0 °C	R: ~60–80% (65% average of 5 cultivars) vs. 10–12% without CA	[97]
		Enc-Deh	0.75 M sucrose in beads → 0.75 M sucrose for 18 h	R: ~55–80% (65% average of 5 cultivars) vs. 5–20% without CA	
*Mentha x piperita* (3 accessions), *M.* × *villosa* (3 accessions), *M. spicata* (2 accessions)	25 °C/−1 °C, 16 h light/8 h darkness for 4 weeks or varying durations	DV	0.3 M sucrose 20–24 h → 2 M glycerol + 0.4 M sucrose for 2 h → PVS2 for 20 min	R: 57–86% vs. 22–53% without CA	[96]
*Pyrus koehnei*, *P. communis*, *P. commu-* *nis* × *P. pyrifolia*, *P. pashia*, *P cordata* (8 genotypes)	22 °C/−1 °C 8 h light/16 h darkness for 1–12 weeks	CF	medium with 0.35% agar/ 0.185 gelrite + 5% DMSO for 48 h under CA condition → 10% PEG8000+ 10% glucose + 10% DMSO 30 min then 30 min at 0 °C	R: ~65–100% vs. ~2–16% of non-CA shoot tips R:~17% vs. ~2% for 1 genotype, genotype-dependent effect of CA	[91]
*Rubus spectabilis*, *R. idaeus*, *Rubus* spp. (2 accessions)	22 °C/−1 °C 8 h light/16 h darkness for 1 week	CF	5% DMSO for 48 h → 10% PEG8000+ 10% glucose + 10% DMSO for 1 h at −1 °C	S: 51–67% vs. 18–41% without CA, genotype-dependent effect of CA, one accession non-responsive	[87]
*Solanum* spp. Method applied to over 4000 accessions	7 ± 2 °C for 2–3 weeks (10–20 μmol m^−2^ s^−1^ for 16 h photoperiod	DV	2 M glycerol + 0.4 M sucrose 20 min → PVS2 at 0 °C for 50 min	R: average 63.5% (20–100% range within species/subspecies)	[99,100]
*Syringa vulgaris* (2 varieties)	8 °C 16 h photoperiod for 2 weeks modified macronutrients + 6% sucrose + 0.2 mg L^−1^ BA + 1.0 mg L^−1^ PBZ	Prec-deh	273.84 g L^−1^ sucrose + 10 g L^−1^ agar at 0–2 °C for 48 h → air dehydration	R: 62–87%	[118]
*Ullucus tuberosus*	5 °C for 3–4 weeks	D-cryoplate	0.3 M sucrose 16 h → 0.4 M sucrose in beads → 2.0 M glycerol + 1.0 M sucrose for 90 min	R: 73–97% (average 90%) for 11 genotypes	[119]
*Vaccinium uliqinosum*, *V. ovatum*, *V. corymbosum*	22 °C/−1 °C 8 h light/16 h darkness for 3, 5, or 7 weeks	CF	Preculture with 5% DMSO for 48 h → 10% PEG8000+ 10% glucose + 10% DMSO on ice, then 30 min at −1 °C	S: 58% vs. 6% without CA for *V. corymbosum*, genotype-dependent effect of CA, some accessions non-responsive	[89]

* Vit, vitrification; DV, droplet-vitrification; Enc-deh, encapsulation-dehydration; Enc-vit, encapsulation-vitrification; Prec-deh, preculture-dehydration; CF, controlled (slow) freezing. ** Conditions used to achieve maximum regrowth: DMSO, dimethyl sulfoxide; PEG, polyethylene glycol; PVS2: 30% glycerol + 15% DMSO + 15% EG 15.0 + 13.7% sucrose; PVS3: 30% glycerol + 30% sucrose. Treatments were performed at room temperature (23 to 25 °C) unless otherwise stated. *** Best regrowth reported or average regrowth among genotypes.

### 3.2. Sucrose Preconditioning of Donor Plants

With the development of vitrification- and encapsulation-based cryopreservation methods, CA could be replaced by preconditioning donor plant material with elevated sucrose or other osmolites, such as sorbitol [120]. This strategy was further explored (Table 4), and the effects of sucrose were compared with those of CA across several crops.

With more detail, the sucrose treatment as an alternative to CA was explored for potato. A 2-week preconditioning of potato donor plants with 0.3 M sucrose revealed genotype-specific responses in regrowth, sugar accumulation, and protein abundance [102]. Compared to CA, sucrose pretreatment led to greater accumulation of rhamnose, arabinose, and stachyose in most genotypes. Oligosaccharides, such as stachyose, accumulate under drought stress; they contribute to osmotic adjustment, stabilize membranes and proteins, and may act as antioxidants [103]. Proteome analysis revealed distinct changes in protein abundance in response to sucrose pretreatment compared with CA [102]. In the cultivar ‘Désirée’, incubation of donor plants in 2 M sucrose for 5 days followed by a 1-day shoot tip preculture in 0.7 M sucrose improved regeneration after cryopreservation [121]. Regrowth increased from 50% to 80% after donor plants were preconditioned with 0.055, 0.11, or 0.22 M sorbitol for 21 days, followed by DV [122]. The effect of exogenous sorbitol on carbohydrate and polyol concentrations in plants was concentration-dependent. Trehalose and endogenous sorbitol increased exponentially with the pretreatment molarity, while levels of sucrose, glucose, fructose, mannitol, arabinose, galactinol, melibiose, and stachyose increased up to 0.11 M sorbitol [122].

Pretreatment of donor plants with 0.3 M sucrose for 15 or 22 days effectively improved post-cryopreservation regrowth of shoot tips for *Agave sobria* (to 87%, [123]) and *Sechium edule* (to 38%, [124]). In *Byrsonima intermedia*, a woody tropical medicinal plant from the Brazilian Cerrado, preconditioning donor plants with 0.3 M sucrose or a combination of sucrose and sorbitol was effective. Still, it could be replaced with preculture of excised shoot tips in 0.3 M sucrose for 24 h [125]. In three taro cultivars, shoot tips from plants pretreated with 0.18 or 0.26 M sucrose for 7 weeks showed better regrowth after cryopreservation (6.7–30%) than at higher sucrose concentrations or shorter pretreatments [126].

In *Gerbera jamesonii*, the cultivation of donor plants was modified by adding a 90% *w*/*v* sucrose stock solution to the gelled medium in culture vessels, resulting in a final concentration of 7.5%, 4 days before shoot tip excision and cryopreservation using DV. This change, combined with recovery on an ammonium-free medium, resulted in 25% regrowth after cryopreservation, compared with nearly zero without sucrose preconditioning [62].

An interesting study was performed on shoot tips of the *Ananas comosus* cultivar cryopreserved by vitrification. The authors studied the effects of short-term (0–7 days), mid-term (15 and 30 days), and long-term (up to 150 days) preconditioning of donor plantlets with varying sucrose concentrations (0.2, 0.3, 0.4, 0.5, or 0.6 M). Among the short-term preconditioning treatments, the highest post-LN survival (66.7%) was achieved after 5-day preconditioning with 0.2 M sucrose, followed by 3 h of dehydration in PVS2. Survival increased gradually with increasing preconditioning duration of donor plants and reached 100% after 135 days with 0.2 M sucrose and 2 h PVS2 exposure [127].

In vitro-grown date palm (*Phoenix dactylifera*) leaves showing bud initiation were cultured with 180 g L^−1^ sucrose or incubated at 4 °C for 2 d before being cryopreserved using three methods: vitrification, DV, and encapsulation-vitrification. These preconditioning treatments equally improved regrowth after cryopreservation and increased proline content in explants [128]. In duckweed, an aquatic flowering plant, precultivation in a medium containing 0.4 M sucrose for 6 days provided better regrowth post-cryopreservation than CA (4 °C, darkness) or a 25 °C/−1 °C 16/8 h light/dark cycle [129].

**Table 4:** Preconditioning donor plants with high concentrations of sucrose or sorbitol before shoot tip cryopreservation.

Species	Donor Plant Treatment	Cryopreservation Method *	Major CPA Used **	Regeneration after cryopreservation (R) ***	Reference
*Ananas comosus* cv. Tainung No. 16	0.2 M sucrose for 135 days	Vit	PVS2	R: 100%	[127]
*Byrsonima intermedia*	0.21 M sorbitol + 0.09 M sucrose for 15 days	DV	PVS2	R: 67% vs. 43% in control	[125]
	0.3 M sucrose for 15 days	DV	PVS2	R: 11% vs. 43–95% in control (negative impact)	
*Colocasia esculenta* var. *esculenta*	0.18 or 0.26 M sucrose for 7 weeks	Vit	PVS2	R: 6.7–30% vs. 0% in control, genotype-dependent effect of sucrose pretreatment of donor plants	[126]
*Gerbera jamesonii*	7.5% (0.22 M) sucrose for 4 days	DV	A3-80%	R: 25% vs. 0% in control	[62]
*Sechium edule* (3 cultivars)	0.3 M sucrose for 22 days	Vit	PVS2	R: 17–38%	[124]
*Solanum tuberosum* cv. Desiree	0.055 M, 0.11 M, and 0.22 M sorbitol for 21 days	DV	PVS2	R: 80% vs. 50% in control	[122]
*Solanum commersonii*, *S. juzepcukii*, *S. ajanhuiri*, *S. tuberosum* cv. Desiree	0.3 M sucrose for 2 weeks	DV	PVS2	R: 0–70%, genotype-specific response to sucrose pretreatment	[102]
*Phoenix dactylifera*, in vitro-grown leaves with bud initiation	180 g L^−1^ (0.53 M) sucrose for 2 days	DV Vit Enc-Vit	PVS2	R: DV—60% Vit—26.7% Enc-Vit—40%	[128]

* Vit, vitrification; DV, droplet-vitrification; Enc-vit, encapsulation-vitrification. ** CPA; cryoprotectant mixture; PVS2, 30% glycerol + 15% DMSO + 15% EG 15.0 + 13.7% sucrose; PVS3, 30% glycerol + 30% sucrose; A3-80%, 33.3% glycerol + 13.3% DMSO + 13.3% EG + 20.1% sucrose. *** Best regrowth reported or average regrowth among genotypes.

In summary, sucrose pretreatment induces beneficial osmotic stress on donor plants and facilitates their metabolic “reprogramming,” preparing the shoot tips for the osmotic stress to come. In contrast to CA, this method applies to tropical crops, e.g., *Agave*, pineapple, taro, and others, for which CA is not suitable. As in CA, the efficiency of sucrose preconditioning may be genotype-dependent. Sucrose pretreatment showed promising results when combined with modern DV, vitrification, cryoplate, and encapsulation-based methods. It is most commonly combined with PVS2 or other four-component vitrification solutions (Table 3), while its effectiveness with PVS3 could not be assessed from the literature.

The effective sucrose concentration, as well as pretreatment duration, remains strictly relative to plant sensitivity to osmotic stress. For example, plants of *Ananas comosus*, which is relatively osmotic-tolerant, can withstand pretreatment with 0.2 M sucrose for up to 135 days. In contrast, *Gerbera jamesonii*, which is very sensitive to both osmotic and chemical stresses, tolerates only a few days of pretreatment at this sucrose concentration. However, most species’ tolerance lies between these two extremes, with good results observed after 2–3 weeks of preconditioning with 0.3 M sucrose.

### 3.3. Growth Regulators and Signaling Molecules During the Preconditioning of Donor Plants

Various combinations of growth regulators, mostly auxins, cytokinins, and gibberellins, are commonly used in plant tissue culture to support shoot development, multiplication, and growth. Other growth regulators and signaling molecules, such as abscisic acid (ABA), jasmonic acid (JA), or salicylic acid (SA), are less common in routine tissue culture but are central to signaling cascades triggered by abiotic stresses, particularly cold and dehydration. A complex interplay among JA, ABA, and SA was demonstrated in plants under cold or osmotic stress, or their combination [103,130,131,132]. Both JA- and ABA-signaling pathways have been linked to cold- and drought-tolerance in plants [130]. JA could enhance cold tolerance by regulating the ICE-CBF/DREB1 cascade, whereas components of ABA signaling are essential for COR gene expression [103]. These two growth regulators act synergistically or antagonistically to fine-tune the cold adaptation process [103]. SA accumulation suppresses plant growth under chilling or cold stress; moreover, exogenous application of SA improves the cold tolerance of several crops, including potato, rice, cucumber, and maize [103]. In addition to ABA, SA regulates drought responses [133]. However, the effects of all the aforementioned growth regulators were reported to be dose-dependent, with high concentrations having the opposite effect, making plants more sensitive to stress-induced damage [130,133]. These growth regulators were investigated as supplements to donor plant pretreatment (Table 5), yielding interesting findings.

Jasmonic acid

Preconditioning pineapple (*Ananas comosus* var. *comosus* ‘MD-2’) donor plants with 1 mg L^−1^ JA for 45 days, combined with DV cryopreservation using PVS3, dramatically increased shoot tip survival (from 74.6 to 98.12%) and regeneration (from 72 to 97.12%) and enhanced the morphological traits (plant length, leaf width, and length) of the regenerated plants. This beneficial effect was confirmed during the acclimatization of plants derived from the cryopreserved shoot tips and validated across nine other pineapple cultivars [134].

Salicylic acid

Preconditioning stock shoots of raspberry, *Rubus idaeus*, with SA (0.05–0.1 mM) for 4 weeks markedly increased their survival after 4 weeks of thermotherapy (to 80%) and enhanced regrowth of cryopreserved shoot tips (to 33%) [135].

In *Vitis*, where the CA of donor plants was not effective [136], plantlet preconditioning with SA was tested. Among varying SA concentrations (0, 0.1, 0.5, and 1 mM) applied for 2 weeks, 0.1 mM SA provided the highest post-cryogenic regeneration (7–40% for six accessions) following the DV protocol with PVS2 [137]. In a study by Bettoni et al. [136], nodal segments were preconditioned for 2 weeks with 0.2 mg L^−1^ BA, 0.1 mM SA, 1 mM ascorbic acid, and 1 mM glutathione (reduced form) before explant excision. This treatment, combined with an optimized PVS2-based DV protocol, resulted in post-cryopreservation survival exceeding 40% across 12 *Vitis* species [136]. Later, Bi et al. [138] reported shoot regrowth from over 60% of cryopreserved *Vitis* shoot tips following a similar protocol. By contrast, in *Vitis vinifera* cv. Portan, cultivation of single-node segments on medium with 50, 500, or 2000 μM L-proline for 2 weeks did not improve shoot tip regrowth following DV using PVS2 [139].

In tea (*Camellia sinensis*), Sharma and Mukherjee [140] investigated various factors, including CA, ABA, increased sucrose, and proline, for donor plants pretreatment. ABA and proline, applied alone or in combination, significantly enhanced shoot tip regrowth following vitrification and V-cryoplate methods. Interestingly, sucrose preconditioning of donor plants (40, 50, and 60 g L^−1^) for one month gave no positive effect on regrowth, indicating the high potential sensitivity of tea to osmotic stress.

Abscisic acid

In *Rubus* spp., ABA pretreatment alone did not improve the survival of cryopreserved shoot tips, but it showed a strongly genotype-dependent effect when combined with CA [88]. Ryynänen and Häggman [113] applied ABA during CA of donor shoot cultures in *Betula pendula*, although the impact of ABA alone was not investigated.

Bruňáková et al. [141] provided insights into the effects of prolonged ABA preconditioning compared to CA on *Hypericum perforatum* shoot tips. Shoot tips were pretreated in a liquid medium supplemented with 0.076 µM ABA and agitated for 3, 7, or 10 days. Alternatively, CA at stepwise decreasing temperatures was applied for 9 weeks. ABA preconditioning significantly reduced both total and freezable water content in the shoot tips, with the effect intensifying over the 10 days. When subsequently loaded and exposed to PVS3, shoot tips pretreated with ABA for 10 days contained less freezable water across all three genotypes and less total water in two of the three genotypes during a 60–240-min dehydration period, compared to CA shoot tips. Following cryopreservation, the recovery of ABA-pretreated shoot tips ranged from 59.3% to 71.3% after PVS3 treatment (180 min) [141].

In *Eucalyptus grandis*, a 5-day ABA (5 mg L^−1^) pretreatment of shoot clusters notably improved the tolerance of excised buds to rapid desiccation and preculture with high sucrose (0.4 to 1.0 M) and glycerol concentrations. Nevertheless, successful cryopreservation has not been reported [142,143].

Common tissue culture growth regulators

The effects of the growth regulators commonly used in tissue culture were also considered during the pretreatment phase. For example, Marković et al. [144] found that culturing *Vitis* microcuttings on a cytokinin-supplemented medium improved regrowth following DV compared to a cytokine-free medium, with BA and zeatin riboside being equally effective. In *Holostemma annulare*, indole-3-butyric acid (IBA) in the preconditioning medium for donor plants had little effect on cryopreservation success, regardless of treatment duration (2 or 30 days) and ammonium concentration, and exerted only a minor influence on tissue water content [145]. In *Rubus idaeus*, the effects of growth regulators benzyl adenine (BA) and thidiazuron (TDZ) varied with the carbon source and cryopreservation method used. BA showed better results with controlled freezing, wherea s TDZ was effective with quick freezing, resulting in 70% regrowth [146]. Paclobutrazol (PBZ), a triazole-type growth retardant known to inhibit the biosynthesis of gibberellins and modulate the biosynthesis or metabolism of indole-3-acetic acid (IAA), cytokinins, ethylene, ABA, and polyamines, was tested for cryopreservation of *Syringa vulgaris* in combinations with TDZ and BA. A modified preculture-desiccation method resulted in 62–87% post-cryopreservation regrowth [118].

Other authors emphasized the need to completely remove growth regulators from the donor plant culture medium to improve vigor. For example, in *Cirsium setidens*, a wild Korean species, the regrowth of cryopreserved shoot tips increased from the randomly observed 20–25% to over 36% by transferring individual plants from a multiplication medium containing 1.5 mg L^−1^ BA to a medium without growth regulators for 3 to 6 weeks [62].

In summary, the use of specific growth regulators during donor plant preconditioning can significantly improve regrowth in some species. However, this appears to be a targeted strategy rather than a routine practice. ABA has the potential to reduce freezable water and induce tolerance to osmotic stress imposed by PVS3 and PVS2, as well as rapid dehydration. JA and SA are effective in inducing stress tolerance in species in which CA fails to elicit the desired response. For some species, particularly those propagated with high cytokinin concentrations, preconditioning donor material on a medium devoid of growth regulators may be beneficial.

**Table 5:** Preconditioning of donor plants with specific signaling molecules, growth regulators, antioxidants, or on an ammonium-modified nutrient medium before shoot tip cryopreservation.

Species	Donor Plant Treatment	Cryopreservation Method *	Major CPA Used **	Regeneration after cryopreservation (R) ***	Reference
*Camellia sinensis*	10 mg L^−1^ ABA + 3 mg L^−1^ proline for 30 days	Vit V-cryoplate	PVS2	R: Vit—60.5% V-cryoplate—75.6%	[140]
*Ananas comosus* var. comosus ‘MD-2’	1 mg L^−1^ jasmonic acid for 45 days	Enc-vit	PVS3	R: 97% vs. 72.35% in control	[134]
*Holostemma annulare*	2.85 µM IBA for 2 or 30 days	Enc-deh	-	R: 6–58% depending on medium composition, but no effect of IBA	[145]
*Rubus idaeus*, genotype Z-13	0.01–0.1 mM salicylic acid for 4 weeks	Enc-vit	PVS2	R: 28–33% vs. 12% in control	[135]
*Vitis* spp. (9 or 12 species)	0.2 mg L^−1^ BA + 0.1 mM salicylic acid + 1 mM ascorbic acid + and 1 mM glutathione (reduced form)	DV	PVS2	R: 35–72 (12 species) R: 24–43% (averaged 35%) (nine species)	[68,136]
*Vitis* spp. (6 accessions)	0.1 mM salicylic acid for 2 weeks	DV	PVS2	R: 7–40% vs. 0–13% in control without SA and preculture of explants with sucrose	[137]
*Dahlia* spp.	30 mg L^−1^ silver nitrate for 6 weeks	DV	PVS2	R: 44.44% average for six accessions, regrowth is stable across 6 years of cryobanking	[147]
*Solanum tuberosum* (2 clones)	1 or 5 mM H_2_O_2_ for 1 h	D-cryoplate	-	R: 28–37% vs. 10–17% in control for two clones	[148]

* Vit, vitrification; DV, droplet-vitrification; Enc-deh, encapsulation-dehydration; Enc-vit, encapsulation-vitrification. ** CPA; cryoprotectant mixture; PVS2, 30% glycerol + 15% DMSO + 15% EG 15.0 + 13.7% sucrose; PVS3, 30% glycerol + 30% sucrose; A3-80%, 33.3% glycerol + 13.3% DMSO + 13.3% EG + 20.1% sucrose. *** Best regrowth reported or average regrowth among genotypes.

### 3.4. Oxidative Stress: The Role of Ammonium Nitrate and Hydrogen Peroxide in Donor Plant Preconditioning

Reducing, omitting, or substituting ammonium at various stages of the cryopreservation protocol is a valid and frequently used strategy that helps mitigate cryopreservation-induced oxidative stress in plant materials and enhance regrowth following cryopreservation [41,52]. However, only a few studies have investigated the role of ammonium in donor plant preconditioning.

In silver birch cryopreservation, the ammonium in the medium used for donor plant CA and shoot tip recovery was entirely replaced with KNO_3_. This change resulted in a substantial increase in post-cryopreservation regrowth, ranging from 15% to 88.3% across genotypes, compared with low regrowth in ammonium-containing variants [113]. Later studies demonstrated that ammonium substitution was most effective in improving regrowth of shoot tips obtained from old (55 months and older) cultures but had a negligible effect on shoot tips excised from younger (less than 20 months old) cultures [86].

For *Agave* cryopreservation [149], donor materials were cultured for 90 days on semi-solid MS medium without growth regulators, with NH_4_NO_3_ reduced to 5 mM. Shoot tips were then excised and cryopreserved using the DV method with PVS2. After cryopreservation, regrowth ranged from 76% to 97% across the seven species [149].

For cryopreservation of potato in the Czech cryobank, in vitro plants were cultured on semi-solid MS medium modified to lower nitrogen levels: 25% NH_4_NO_3_ and 50% KNO_3_ relative to the original formulation [150]. Four-day-old nodal cuttings were then treated with 2 M sucrose for the next five days, followed by shoot tip excision and cryopreservation via the preculture-desiccation method. Decruse et al. [145] investigated the effects of MS medium containing 0.0, 3.75, and 20.6 mM NH_4_NO_3,_ as well as a modified MS medium containing 2.6 mM NH_4_H_2_PO_4_ throughout the encapsulation-dehydration cryopreservation protocol, including donor plant preconditioning (2 or 30 days) for *Holostemma annulare*. In all treatments, 30-day preconditioning generally improved post-LN regrowth compared to a short 2-day treatment. Under long preconditioning of donor plants, the medium without ammonium nitrate yielded the highest direct regeneration after desiccation (81.7%) and cryopreservation (48.2%). Medium with NH_4_H_2_PO_4_ produced a similar response. Increasing ammonium nitrate concentration progressively reduced regeneration to 8–23% and caused callus formation on cryopreserved shoot tips [145].

Recently, an interesting case was reported for potato, in which single-node segments of two clones were pretreated with hydrogen peroxide (0, 0.1, 1, 2.5, 5, 10 mM) for 1 h, followed by 30 d of culture under standard conditions. Axillary buds subjected to 1 and 5 mM treatments were excised and biochemically analyzed or cryopreserved using the D-cryoplate method [148]. The H_2_O_2_ pretreatment had a prolonged, apparent effect on plant growth, reducing both plant height and fresh biomass gain in a concentration-dependent manner. It also enhanced stress tolerance, decreased the water potential of buds, and improved regrowth after cryopreservation, increasing it from ~10–17% to ~28–37%. These changes correlated with shifts in antioxidant enzyme activity in H_2_O_2_-pretreated buds, both before and after cryopreservation. In particular, CAT and POX activities after cryopreservation were higher in buds excised from H_2_O_2_-exposed plants [148]_._ Hydrogen peroxide content in pretreated buds was elevated before cryopreservation but dropped significantly after cryopreservation compared with control buds, indicating greater activity of the ROS-scavenging systems.

Despite very few studies mentioned above, the role of ammonium during pre-cryopreservation steps, such as donor plant preconditioning, remains poorly defined and is not linked to any specific cryopreservation method. Based on the available literature, preconditioning in ammonium-free or ammonium-substituted media may be most effective when followed by regrowth on an ammonium-free medium. This approach could be particularly beneficial for species that are highly sensitive to both osmotic and chemical stress and remain unresponsive to common cryopreservation protocols.

Hydrogen peroxide treatment of donor plants recently emerged as a potential strategy to improve the cryopreservation tolerance of genotypes that do not respond well to conventional cryogenic protocols.

## 4. In Vitro Cultivation Factors

### 4.1. Donor Plant Age

Explants excised from cultures at optimal age usually exhibit greater tolerance to cryopreservation procedures, although the age effect is species- and explant-dependent.

The importance of the physiological state of donor plant material was demonstrated in the experiments with cassava. Shoot tips excised from 21-day-old node sections produced significantly higher survival after cryopreservation via encapsulation-vitrification (82%) than those cultured for shorter periods (7–14 days, 38–44%) or longer periods (28 days, 64%) [151].

Gowthami et al. [152] reviewed critical factors for successful cryopreservation in ornamental plants, highlighting donor culture preconditioning and culture age as particularly important. For example, in *Dahlia*, a popular ornamental plant, regrowth depended critically on donor age: shoot tips from 6- to 8-week-old plants showed notably higher regrowth (23%) than those from younger (4-week-old; 13%) or older (12-week-old; 7%) plants [147]. The optimal age of *Chrysanthemum morifolium* donor plants was 4–5.5 weeks for apical shoot tips and 7 weeks for axillary shoot tips. Both explant types exhibited high regrowth post-cryopreservation (82% and 85%) [153].

In three taro cultivars, in vitro donor plant age (1–4 months) had no effect on shoot tip regrowth after PVS2 treatment (12 min) but significantly influenced regrowth post-cryopreservation. In all cultivars, the highest recovery (14–29%) occurred in shoot tips excised from 3-month-old plants [126].

In the study by Keller on different *Allium* clones, younger cultures that had undergone 3–4 transfers generally responded better to PVS3 treatment and vitrification cryopreservation than 3.5-year-old cultures [114]. However, in some clones, regrowth of older materials could be improved after CA. In silver birch, the recovery of shoot tips derived from young (less than 20-month-old) cultures was significantly better than that from 55-month-old cultures (average 38% vs. 15% of five genotypes). However, the effect of age was significant only in one genotype [86].

For three apple cultivars, extending the donor plant period without subculture to 12 and 29 weeks (including CA) reduced shoot-tip water content by 10% and 20%, respectively, compared with 6-week subculture. Shoot tips from 12-week-old plants with water content reduced to 68% showed much higher regrowth than those excised from younger cultures using three methods: vitrification, programmed freezing, and encapsulation-dehydration [90]. In the study by Condello et al. [154], improved regrowth was observed when apple axillary buds were excised from aged mother-plants in comparison to those excised from plantlets that were regularly subcultured. The highest regrowth was obtained with non-preconditioned 4-month-old in vitro shoots.

Guerra et al. [155] assessed how the culture period of donor plants (30, 45, 60 days) influenced the survival of pineapple shoot tips cryopreserved via DV with PVS2. The study, performed across four accessions and two hybrids, revealed a significant genotype-by-culture-by-time interaction. Moreover, regeneration success was highly genotype-dependent. Two genotypes showed peak regeneration (90–95%) from 30-day-old donor plants, while another achieved 100% regeneration from plants aged 45–60 days. Two other genotypes failed to regenerate or showed consistently low rates (17–20%), regardless of plant age. These results highlighted the need for genotype-specific protocol adjustment, including optimization of donor plant age [155]. For the cultivars that regenerated best from 30-day-old plants, the relationship between donor plant culture period, shoot tip anatomy, and regeneration was histologically confirmed. Meristematic regions of 30-day-old shoot tips were characterized by isodiametric cells with dense cytoplasm, whereas 45-day-old tips exhibited large vacuoles and intercellular spaces, i.e., traits associated with high water content and freezing injury, linked with low regeneration [155].

In mint, the optimal age of donor plant culture for shoot tip cryopreservation was also genotype-dependent, ranging from 4 to 8 weeks, and even up to 10 weeks [96]. Cutting nodal sections from 3- to 8-week-old mint cultures and culturing them for 2 to 5 days to elongate before shoot tip excision promoted uniform growth and maximized the efficient use of in vitro material [156].

In *Rubus humulifolius*, cryopreservation via DV was most effective with 1-month-old plants (52% regeneration) compared to 2- and 4-month-old plants (30% and 0% regeneration, respectively) [157]. Shoot tips of Yukon Draba (*Draba yukonensis*), a Canadian endemic, showed better regrowth following PVS3 treatment and DV cryopreservation when excised from 2-week-old donor plants than from 1-, 3-, or 4-week-old donor material [158]. Meanwhile, for Hill’s thistle (*Cirsium hillii*), donor plant age (3 to 6 weeks) did not significantly affect shoot tip regrowth, which remained above 90% both before and after cryopreservation via PVS3-based DV [159].

### 4.2. Physical Conditions: Light, Aeration, Planting Density, and Medium Strength

Apart from temperature and photoperiod during the CA, the effects of physical conditions during donor plant cultivation have rarely been explored.

Using cultivated and wild potato genotypes, Yoon et al. 2006 [160] studied the influence of donor plants’ subculture conditions, i.e., subculture duration, light intensity, aeration, and planting density, on shoot tip regrowth following DV cryopreservation. These parameters significantly affected the regrowth of both non-cryopreserved and cryopreserved shoot tips. The optimal subculture duration was 5 or 7, depending on the cultivar. Aeration was the most critical parameter among those tested. The highest regrowth (70.9%) was observed with a combination of high light intensity (130 µmol m^−2^ s^−1^), ventilation of culture vessels (sealed with one round of cling film), and low planting density (seven node cuttings per culture vessel). This was statistically different from the 32–47% regrowth of shoot tips excised from plants cultured in air-tight vessels (sealed by two rounds with Parafilm and three rounds with cling film). Under low illumination (20 µmol m^−2^ s^−1^), planting density was a significant factor, with low density yielding 65% survival vs. 44% at high-density culture (17–24 node cuttings per vessel) [160]. On the other hand, Vollmer et al. [100] reported the use of a high-density (80–90 explants per Petri dish) culture of uninodal stem segments in a routine protocol for potato cryopreservation without adversely affecting regeneration success.

For several crops, such as *Fragaria* spp. and *Syringa* spp., relatively high agar concentrations (0.85–1.0%) were used in the medium for plant preconditioning [92,118]. On the contrary, other authors found that adding an overlay of liquid medium on top of the gelled medium during donor plant propagation may result in notable improvements in vigor and subsequent response to cryopreservation. This effect was reported for *Pogostemon yatabeanus* [161], as well as for *Chrysanthemum* and *Aster* spp. [56,62], and *Penthorum chinense* [58].

Although light regime and quality may be critical for in vitro-cultured plants, these factors have been rarely studied during cryopreservation. During CA, low light intensity and a shorter illumination period (8 h light/16 h dark or 12 h light/12 h dark) combined with lower temperature (Table 3) are expected to mimic natural autumn conditions, thereby triggering plant signaling systems for more efficient cold hardening.

The light spectrum, both pre- and post-cryopreservation, may significantly affect shoot tip regrowth, as demonstrated in potato [162,163]. Before shoot tip excision for cryopreservation, potato nodal cuttings were exposed to various light sources for 3.5 weeks; cultivation under blue LEDs resulted in earlier recovery (2 weeks post-cryopreservation), whereas red LEDs had an adverse effect. Post-cryopreservation regrowth for five cultivars ranged from 31–66% for shoot tips from nodal segments cultured under blue light, to 9–48% and 2–26% after white and red light, respectively [164]. Light quality significantly affected gene expression in shoot tips, up- and down-regulating 3284 genes [164]. Interestingly, the light spectral quality treatments did not cause significant changes in either survival or regeneration of non-cryopreserved control shoot tips [165].

### 4.3. Explant Type and Uniformity

The type of explant (shoot tip or nodal bud) and the bud’s position on the stem may significantly influence survival under cryopreservation stress, as they differ in physiological state. In the case of carnation, for example, the lower the position of axillary buds on the stem (starting from the terminal bud), the lower post-cryopreservation regrowth [166]. By contrast, in *Chrysanthemum morifolium* cv. Peak, axillary shoot tips were more sensitive to PVS3-induced osmotic stress than apical shoot tips and required an optimal treatment of 60 min, compared to 90 min for apices [153]. Similarly, in sweet potato, axillary meristems showed higher regrowth after cryopreservation via PVS2-based DV than apical meristems in two of three genotypes [67]. In *Arabidopsis thaliana*, axillary buds tolerated PVS3 treatment better than shoot tips. While shoot tips required a 60-min optimum treatment for 100% regrowth, axillary buds achieved over 90% regrowth even after 180 min of PVS3 exposure and subsequent cryopreservation [167]. In *Vitis*, buds sampled from nodes 3–4 and 6–7 (from the top of the stem) showed greater regrowth than shoot tips [144]. In another study, axillary buds showed higher tolerance to PVS2 than apical shoot tips in six accessions [137].

To reduce this potential heterogeneity, many authors use single-nucleus microcuttings as donor material for shoot tip excision during cryopreservation. This strategy was effective in potato [100], *Vitis* [144], *Chrysanthemum* [56], and other species.

The physiological age and condition of donor plant material are critical factors for successful cryopreservation. Explants excised within a species- and genotype-specific optimal age window (typically at an intermediate stage of maturity) demonstrate significantly higher regrowth after cryopreservation. This optimal state is characterized by favorable anatomical traits, such as cells with dense cytoplasm and lower freezable water content, which confer greater tolerance to dehydration and freezing stresses. However, this optimal window is not universal and must be determined empirically for each plant and explant type. The effect of donor age can vary dramatically across genotypes, and factors such as subculture interval, aeration, and light intensity further modulate the explant’s physiological state. Therefore, standardizing and optimizing donor plant culture conditions is a fundamental prerequisite, not a preliminary step, for developing reliable and efficient cryopreservation protocols. In addition, younger cultures may respond better to cryopreservation than cultures maintained in vitro for several years; hence, rejuvenation of in vitro clones before cryopreservation may be considered for recalcitrant material. Donor plant age may be less critical for species that are inherently tolerant to the osmotic and chemical stresses of cryopreservation.

## 5. Integration of Donor Plant Preconditioning to Modern Cryopreservation Protocols: Conclusion and Future Considerations

In current shoot tip cryopreservation practice, a significant challenge is the high sensitivity of certain species and genotypes to stress induced by concentrated vitrification solutions. This stress, which can be osmotic, chemical, or both, often results in poor regeneration under standard procedures. When stepwise optimization of the preculture or cryoprotectant treatment fails to improve regrowth, it is essential to re-examine the donor plant material from which the shoot tips are excised. In this context, moving forward may require revisiting the methods and practices developed over the past decades to improve the stress tolerance of donor materials and, perhaps, reinventing some of them or creating new ones.

This review began with the hypothesis that specific factors in donor plant preconditioning could be linked to specific cryopreservation methods and to shoot tip tolerance to cryopreservation stress, whether chemical or osmotic. Based on literature analysis, we conclude that CA remains highly effective for controlled freezing and PVS2-based vitrification, i.e., methods that impose prolonged chemical and chilling stress but not rapid, severe dehydration. In contrast, sucrose or sorbitol preconditioning offers a broader, often more practical alternative for inducing osmotic tolerance, proving effective for both temperate and tropical species in a range of methods, including vitrification-, encapsulation-, and cryoplate-based methods. For particularly challenging cases, e.g., for very osmotic-sensitive materials, targeted application of growth regulators (e.g., ABA for dehydration/osmotic stress, JA or SA for stress signaling) or culture medium modifications (e.g., reducing ammonium to mitigate oxidative stress) can provide the necessary physiological adjustments. The newly reported method of a short H_2_O_2_ pretreatment of donor plant material may also become an effective alternative.

The core benefit of these preconditioning treatments is the induction of favorable physiological and anatomical changes in shoot tips: reduced freezable water content, accumulation of protective osmolytes and antioxidants, membrane stabilization, and activation of antioxidant defense systems. These changes collectively enhance material tolerance to severe dehydration and chemical toxicity. Importantly, the age, type, and uniformity of explants, dictated by donor culture conditions (light, aeration, subculture interval), may be critical factors that must be standardized to ensure high regrowth.

**Figure 3:**
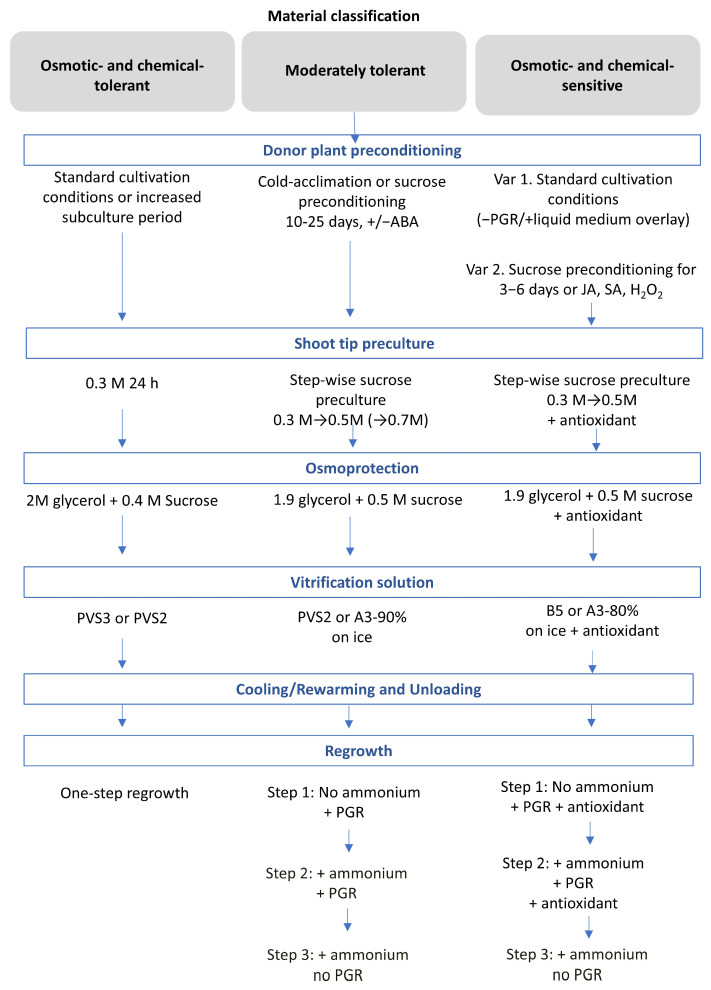
Proposed integration of donor plant preconditioning into shoot tip cryopreservation using the systematic protocol based on material sensitivity/tolerance to stress provoked by the protocol steps. PGR: plant growth regulators.

The proposed integration of donor plant preconditioning into the cryopreservation systematic approach, depending on the plant material category, is illustrated in Figure 3.

A combination of CA and PVS2 in the protocol is often used with inherently chilling-tolerant materials. It therefore usually requires a one-step shoot tip pretreatment before loading for successful cryopreservation. By contrast, for medium-sensitive materials, pretreatment of donor plants with sucrose or signaling molecules for 2 to 4 weeks may be combined with a stepwise preculture of excised shoot tips at increasing sucrose concentrations. For very sensitive materials, donor plant pretreatment on medium without ammonium, use of stress-inducing chemical agents (e.g., H_2_O_2_), or a few-day treatment with sucrose may be used in combination with 0.3 M sucrose preculture of the shoot tips and less concentrated alternative vitrification solutions, such as A3-70% and B5-80%, applied on ice. In this case, applying a stepwise regrowth protocol, starting with an ammonium-free medium and using antioxidants such as ascorbic acid throughout the procedure, may significantly improve regrowth.

In conclusion, preconditioning donor plants, combined with protocol tailoring, may offer new opportunities to improve the cryopreservation of species that are inherently sensitive to osmotic or chemical stress associated with modern cryopreservation protocols.

## Figures and Tables

**Table 1:** Composition of solutions frequently used for preculture, osmoprotection, cryoprotection, and unloading in the droplet-vitrification procedure.

Protocol Stage	Solution *	Composition (%, *w*/*v*) **	Total Concentration (%, *w*/*v*)	References
Explant Preculture	S-10%	S 10.0	10.0	-
	S-17.5%	S 17.5	17.5	-
Osmoprotection	C4-35%	G 17.5 + S 17.5	35.0	[55]
	C6-40%	G 20.0 + S 20.0	40.0	[55]
	C7-32.1%	G 18.4 (2 M) + S 13.7 (0.4 M)	32.1	[55]
Cryoprotection (vitrification solution)	A1-73.7% (PVS2)	G 30.0 + DMSO 15.0 + EG 15.0 + S 13.7	73.7	[38]
	A3-90%	G 37.5 + DMSO 15.0 + EG 15.0 + S 22.5	90.0	[36]
	A3-80%	G 33.3 + DMSO 13.3 + EG 13.3 + S 20.1	80.0	[56]
	A3-70%	G 29.2 + DMSO 11.7 + EG 11.7 + S 17.4	70.0	[57]
	B1-100% (PVS3)	G 50.0 + S 50.0	100.0	[39]
	B5-85%	G 42.5 + S 42.5	85.0	[58]
	B5-80%	G 40.0 + S 40.0	80.0	[36]
Unloading	S-35%	S 35.0	35.0	[59]

* Names of the solutions used in the references. ** G, glycerol; S, sucrose; DMSO, dimethyl sulfoxide; EG, ethylene glycol. All solutions are prepared using the culture medium; pH is adjusted to 5.8 before filter sterilization.

**Table 2:** Tolerance levels of different crops to osmotic (O) and chemical (C) stresses provoked by, respectively, vitrification solutions PVS3 and PVS2 in vitrification-based cryopreservation studies. The tolerance level was determined based on material regrowth after treatment with PVS3 or PVS2 without cryopreservation (LNC), corresponding to the maximum regeneration after cryopreservation (LN). T—tolerant (regeneration above 80%); M—medium (60−80%); S—sensitive (below 60%). Representative examples of crops for each category are given. The full version of the table, including all studies analyzed, is available in the Supplementary Materials (Table S1).

Species	Explant Size	Method *	Preculture → Osmoprotection	Cryoprotection **	LNC Regeneration ***	LN Regeneration	Category	References
Crops tolerant to chemical stress (C-T)								
*Rosa* × *hybrida* (3 varieties)	3~4 mm	DV	S-17.5% (24 h)	PVS2 (20 min, RT)	84~97%	50~61%	C-T	[63]
*Solanum tuberosum*	2 mm	DV	3 weeks at 5 °C → S-15.4% (48 h) → G18.4% + S27.4% (30 min)	PVS2 (50 min, 0 °C)	93%	71%	C-T	[64]
*Ananas comosus* (16 genotypes)	0.5–1 mm	DV	S-10%, solid (48 h)	PVS2 (30~60 min, 0 °C)	93–95%	90–91%	C-T	[65]
*Stevia rebaudiana*	1–1.5 mm	DV	2 weeks at 4 °C → S-17.5% (48 h, 4 °C) → C7-32.1% (20 min)	PVS2 (60 min, 0 °C)	90%	80.0%	C-T	[66]
Crops with medium tolerance to chemical stress (C-M)								
*Ipomoea batatas* (10 varieties)	1 mm	DV	no preculture → C7-32.1% (20 min)	PVS2 (30 min, 0 °C)	67.6	48.1	C-M	[67]
*Vitis* spp. (9 species)	1–1.5 mm	DV	S-3% + antioxidants → S-10% (72 h) → C7-32.1% (20 min)	½ PVS2 (30 min, RT) → PVS2 (75 min, 0 °C)	73%	26%	C-M	[68]
*Citrus limon* (2 varieties)	2.0–2.5 mm	DV	S-10% (48 h) → S-17.5% (16 h) → C4-35% (40 min, RT)	PVS2 (60 min, 0 °C)	76~77%	50.3~53.5%	C-M	[69]
*Malus* × *domestica* ‘Gala Must’	N/R	Vit	S-10% (15 h) → S-25% (5 h) → C7-32.1% (20 min)	A3-90% (50 min, 0 °C)	63.6%	75.0%	C-M	[70]
Crops sensitive to chemical stress (C-S)								
*Prunus cerasifera*	1–2 mm	DV	S-10% (15 h) → S-25% (5 h) → G17.5% + S17.1% (30 min)	A3-90% (10 min, RT)	45.6%	20.0%	C-S	[71]
*Gerbera jamesonii*		DV	S-10% (31 h) → S-17.5% (16 h) → C4-35% (40 min)	VS A3-80% (0 °C, 60 min)	10–25%	10–25%	C-S	[62]
Crops tolerant to osmotic stress (O-T)								
*Lilium* spp.	bulblets, 2 mm	DV	S-10% (31 h) → S-25% (16 h) → C4-35% (40 min)	PVS3 (240 min, RT)	97.7%	87.5%	O-T	[72]
*Allium sativum*	clove apices, 3.5 × 3 mm	DV	S-10% (3 d, solid medium) G18.4% + S20.5% (50 min)	PVS3 (150 min, RT)	97.9%	98.8%	O-T	[36]
*Chrysanthemum morifolium*	1.2–1.5 mm	DV	S-10% (27 h) → S-17.5% (18 h) → S-25% (8 h) → G18.4% + S20.5% (40 min)	PVS3 (60 min, RT)	86.7%	73.1%	O-T	[36]
*Prunus domestica* var. ‘Crvena Ranka’	N/R	V-Cryoplate	S-10% (24 h) → gel → C4-35% (30 min)	PVS3 (60 min, RT)	90.0%	66.7%	O-T	[73]
Crops with medium tolerance to osmotic stress (O-M)								
*Fragaria* × *ananassa* (2 varieties)	2–3 mm	DV	(DV) S-8.6% (24 h) → C4-35% (40 min)	B5-80% (40 min, RT)	73.8~75.7%	50.5~55.6%	O-M	[74]
*Rubus fruticosus*	1–2 mm	DV	S-10% (15 h) → S-25% (5 h) → G17.5% + S17.1% (30 min)	PVS3 (60 min, RT)	77.5%	70.0%	O-M	[71]
Crops sensitive to osmotic stress (O-S)								
*Lithodora rosmarinifolia*	Apical node, 2–3 mm	DV	S-10% (16 h) → S-25% (5 h) → C4-35% (20 min)	PVS3 (60 min, RT)	53%	33%	O-S	[75]
*Freesia hybrida* (2 varieties)	2 × 2 mm	DV	S-10% (31 h) → S-17.5% (16 h) → C4-35% (40 min)	PVS3 (60~120 min, RT)	31~27%	10~22%	O-S	[76]
*Betula lenta*	0.5–1 mm	DV	S-10% (24 h) → C4-35% (20 min)	PVS3 (60 min, RT)	27%	13%	O-S	[60]
*Prunus cerasus* × *P. canescens* ‘Gisela 5′	1–2 mm	DV	S-10% (15 h) → S-25% (5 h) → G17.5% + S17.1% (30 min)	PVS3 (60 min, RT)	36.4%	40.0%	O-S	[77]

* Vit, vitrification; DV, droplet-vitrification; EV, encapsulation-vitrification; ** G, glycerol; DMSO, dimethyl sulfoxide; EG, ethylene glycol; S, sucrose. The composition of some CPA solutions is listed in Table 1. RT, room temperature. *** Mean regrowth of the cryoprotected control (LNC) shoot tips corresponds to the maximum regrowth of cryopreserved (LN) shoot tips.

## Data Availability

During the preparation of this manuscript, the authors used the data from the published sources. No new data were generated.

## Supplementary Materials

The following supporting information can be downloaded at https://www.mdpi.com/article/10.3390/plants15081221/s1; Table S1: Tolerance levels of different crops to osmotic (O) and chemical (C) stresses provoked by, respectively, vitrification solutions PVS3 and PVS2 in vitrification-based cryopreservation studies (full version); Table S2: Use of cold acclimatization in cryopreservation of shoot tips of various crops, horticultural, and model plants (full version) [168,169,170,171,172,173,174,175,176,177,178,179].
