# Supplementary material for: Beyond the Protocol: Revisiting the Critical Role of Donor Plants in Cryopreservation of Economically Important Clonal Crops
Source: Plants (Basel). 2026 Apr 16;15(8):1221. doi: 10.3390/plants15081221 (PMC13120539; doi:10.3390/plants15081221)
Supplement: Supplementary file 1 [file plants-15-01221-s001.zip › plants-4193953-supplementary.pdf]

Supplementary materials to

Beyond the protocol: revisiting the critical role of donor plants in cryopreservation of economically important clonal crops

Elena Popova, Haeng-Hoon Kim

Table S1 (full version of Table 2). Tolerance levels of different crops to osmotic (O) and chemical (C) stresses provoked by, respectively, vitrification solutions PVS3 and PVS2 in vitrification-based cryopreservation studies. Tolerance level was determined based on material regrowth after treatment with PVS3 or PVS2 without cryopreservation (LNC), corresponding to maximum regeneration after cryopreservation (LN). T – tolerant (regeneration above 80%); M – medium (60–80%); S – sensitive (below 60%).

Species	Size, mm	Method*	Preculture → Osmoprotection	Cryo-Protection**	LNC regeneration***	LN regeneration	Category	References
Crops tolerant to chemical stress (C-T)								
Rosa × hybrida L. 3 var.	3~4 mm	DV	S-17.5% (24 h)	PVS2 (20 min, RT)	84~97%	50~61%	C-T	[63]
Fragaria x ananassa	2–3 mm	DV	S-8.6% (24 h)	PVS2 (20 min, RT)	98%	63%	C-T	[168]
Solanum tuberosum	2 mm	DV	3 weeks at 5°C→S-15.4% (48 h)→G18.4% + S27.4% (30 min)	PVS2 (50 min, 0 °C)	93%	71%	C-T	[64]
Prunus cerasus x P. canescens 'Gisela 5'	2 mm	Vit	S-10% (15 h)→ S-17.5% (5 h)→ C7-32.1% (20 min)	A3-90% (30 min, 0 °C)	100%	39–56%	C-T	[77]
Ananas comosus 16 genotypes	0.5–1 mm	DV	S-10%, solid (48 h)	PVS2 (30~60 min, 0 °C)	93-95%	90-91%	C-T	[65]
Actinidia chinensis var. *chinensis* (2 weeks subculture)	0.5–1 mm	DV	S-10% (24 h)→S-25% (24 h)→ C7-32.1% (20 min, 0 °C)	PVS2 (30 min, 0 °C)	96.8%	87.6%	C-T	[95]
Chrysanthemum morifolium	1.5 mm	DV	S-10% (31 h)→S-17.5% (16 h) → C4-35% (40 min)	A3-80% (60 min, ice)	93.0%	86.7%	C-T	[56]
Stevia rebaudiana	1–1.5 mm	DV	2 weeks at 4°C→ S-17.5% (48 h, 4°C)→C7-32.1% (20 min)	PVS2 (60 min, 0 °C)	90% /89%	80.0% /50.0%	C-T	[66]
Prunus insititia "Crvena Ranka"	N/R	DV	(no information on preculture) G17.5% + S17.1% (30 min)	A3-90% (20~30 min, RT)	80.0%	36.4%	C-T	[169]
Crops with medium tolerance to chemical stress (C-M)								
Ipomoea batatas 10 varieties	1 mm	DV	no preculture→C7-32.1% (20 min)	PVS2 (30 min, 0 °C)	67.6	48.1	C-M	[67]
Prunus cerasifera	N/R	V-Cryoplate	S-10% (24 h)→gel→C7-32.1% (30 min)	A3-90% (30 min, RT)	73.4%	56.1%	C-M	[170]
Vitis 9 species	1–1.5 mm	DV	S-3%+antioxidants→solid S-10% (72 h)→C7-32.1% (20 min)	1/2 PVS2 (30 min, RT) → PVS2 (75 min, 0 °C)	73%	26%	C-M	[68]

Citrus limon 2 var.	2.0–2.5 mm	DV	S-10% (48 h)→S-17.5% (16 h) →C4-35% (40 min, RT)	PVS2 (60 min, 0 °C)	76~77%	50.3~53.5%	C-M	[69]
Aster altaicus	1.5 mm	DV	S-10% (55 h)→S-17.5% (17 h) C4-35% (60 min, 0 °C)	A3-80% (60 min, 0 °C)	72%	60%	C-M	[171]
Malus x domestica 'Gala Must'	N/R	Vit	S-10% (15 h)→S-25% (5 h)→ C7-32.1% (20 min)	A3-90% (50 min, 0 °C)	63.6%	75.0%	C-M	[70]
Crops sensitive to chemical stress (C-S)								
Prunus cerasifera	1–2 mm	DV	S-10% (15 h)→S-25% (5 h)→ G17.5%+S17.1% (30 min)	A3-90% (10 min, RT)	45.6%	20.0%	C-S	[71]
Gerbera jamesonii		DV	S-10% (31 h) →S-17.5% (16 h) → C4-35% (40 min)	VS A3-80% (0 °C, 60 min)	10-25%	10-25%	C-S	[62]
Crops tolerant to osmotic stress (O-T)								
Castilleja levisecta	N/R	DV	S-10% (17 h)→S-17.5% (4 h) C4-35% (40 min)	A3-90% (40 min, 0 °C)	80-90%	66%	O-T	[61]
Lilium spp.	1–2 mm	DV	S-10% (31 h)→S-25% (17 h)→C4-35% (40 min)	PVS3 (60 min, RT)	84.0%	65.7%	O-T	[172]
Lilium spp.	bulblets, 2 mm	DV	S-10% (31 h)→S-25% (16 h)→ C4-35% (40 min)	PVS3 (240 min, RT)	97.7%	87.5%	O-T	[72]
Allium sativum	clove apices, 3.5 × 3 mm	DV	S-10% (3 d, Solid medium) G18.4% + S20.5% (50 min)	PVS3 (150 min, RT)	97.9%	98.8%	O-T	[36]
Chrysanthemum morifolium	1.2–1.5 mm	DV	S-10% (27 h)→S-17.5% (18 h) →S-25% (8 h)→G18.4% + S20.5% (40 min)	PVS3 (60 min, RT)	86.7%	73.1%	O-T	[36]
Rubus fruticosus	1–2 mm	DV	S-10% (15 h)→S-25% (5 h)→ C4-35% (30 min)	PVS3 (40 min, RT)	90%	85%	O-T	[173]
Prunus domestica var. 'Crvena Rankat'	N/R	V-Cryoplate	S-10% (24 h)→gel→C4-35% (30 min)	PVS3 (60 min, RT)	90.0%	66.7%	O-T	[73]
Platycodon grandiflorum	1 mm	V-Cryoplate	solid S-10% (16 h)→bead→ G18.4% + S34.2% (30 min)	PVS3 (50 min, RT)	88%	83.8%	O-T	[174]
Crops with medium tolerance to osmotic stress (O-M)								
Fragaria x ananassa 2 var.	2–3 mm	DV	(DV) S-8.6% (24 h)→C4-35% (40 min)	B5-80% (40 min, RT)	73.8~75.7%	50.5~55.6%	O-M	[74]
Rubus fruticisus	1–2 mm	DV	S-10% (15 h)→S-25% (5 h)→ G17.5%+S17.1% (30 min)	PVS3 (60 min, RT)	77.5%	70.0%	O-M	[71]
Crops sensitive to osmotic stress (O-S)								
Lithodora rosmarinifolia	Apical node, 2–3 mm	DV	S-10% (16 h)→S-25% (5 h)→ C4-35% (20 min)	PVS3 (60 min, RT)	53%	33%	O-S	[75]
Freesia hybrida 2 var.	2 × 2 mm	DV	S-10% (31 h)→S-17.5% (16 h) →C4-35% (40 min)	PVS3 (60~120 min, RT)	31~27%	10~22%	O-S	[76]
Betula lenta	0.5–1 mm	DV	S-10% (24 h)→C4-35% (20 min)	PVS3 (60 min, RT)	27%	13%	O-S	[60]
Prunus cerasus x P. canescens 'Gisela 5'	1–2 mm	DV	S-10% (15 h)→S-25% (5 h)→ G17.5% + S17.1% (30 min)	PVS3 (60 min, RT)	36.4%	40.0%	O-S	[77]
Prunus domestica "Sitnica"	N/R	DV	(no information on preculture) G17.5% + S17.1% (30 min)	PVS3 (90 min, RT)	30.0%	18.2%	O-S	[169]

*Vit, vitrification; DV, droplet vitrification; EV, encapsulation–vitrification;

** G, glycerol; DMSO, dimethyl sulfoxide; EG, ethylene glycol; S, sucrose. The composition of some CPA solutions is listed in Table 1. RT, room temperature;

***Mean regrowth of the cryoprotected control (LNC) shoot tips corresponding to maximum regrowth of cryopreserved (LN) shoot tips;

Table S2 (full version of Table 3). Use of cold acclimatization in cryopreservation of shoot tips of various crops, horticultural, and model plants.

Species	CA treatment	Method*	Cryopreservation procedure**	Survival (S) or regeneration (R)***	Reference
Actinidia chinensis var. *chinensis* 'Hort16A'	4 °C for 2 weeks, 10-h photo-period (25 µmol s−1 m−2)	DV	0.25 M–0.5 M– 0.75 M–1.0 M sucrose + 0.4 mM ascorbic acid (1 day each, 4 °C)->2 M glycerol + 0.4 M sucrose 20 min 0 °C →PVS2 0 °C 60 min	R: 40% vs <10% without CA and explant preculture with sucrose	[42]
Allium, 6 genotypes	25/−1 °C under 16 h photoperiod (60-80 µmol/m2s) or 2 °C same light for 10-12 weeks	Vit	0.3 M sucrose 1 day →0.4 M sucrose + 2 M glycerol for 20 min →PVS3 for 120 min	R: ~46-100% vs ~6-40% depending on genotype	[114]
Arabidopsis thaliana ecotype 'Columbia'	4 °C 8 h light/16 h dark for 8 or 18 d	DV	½ MS with 0.3 M sucrose 20-24 h →2 M glycerol + 0.4 M sucrose 20 min →PVS2 at 0 °C for 60 min or PVS3 at 22 °C for 60 min	R-PVS2: ~85% vs ~43% without CA<br>R-PVS3: 100%	[167]
		CF	5% PEG8000+5% glucose+5% DMSO+0.4M sucrose for 30 min → 10% PEG8000+10% glucose+10% DMSO+0.4M sucrose for 30 min at 22 °C	R: ~85% (depending on termination temperature)	[167]
Betula pendula	5 °C 8 h/16 h (light: dark) for 3 weeks	CF	5% DMSO 72 h →10% PEG+10% glucose+10% DMSO 30 min+30 min at 0 °C	R: 19-27% vs ≤1% without CA for 4 lines, genotype-dependent response	[86]
Betula pendula	+5 °C 8/16h light/dark + 100 µM L^{-1} ABA for 28 days<br>NH_4N0_3 and $Ca(N0)_3$ were substituted by $KN0_3$ (10 mmol L^{-1})	CF	0.5% DMSO + 100 µM/L ABA for 72 h (5 °C 8/16h light/dark) →10% PEG+10% glucose+10% DMSO 30 min+30 min at 0 °C	R: average 58.3 % (15-88.3% for 4 genotypes, genotype-dependent response)	[113]
Chrysanthemum morifolium cv. shuhounochikara	10°C 1 8 h light/16 h dark for 3 weeks	Enc-deh	0.3 M sucrose at 5 °C in dark 3 days →2 M glycerol + 0.4 M sucrose in beads 1 h	R: 85%	[29]
		Vit	0.3 M sucrose at 5°C in darkness 3 days →2 M glycerol + 0.4 M sucrose 20 min →PVS2 20 min	R: 85%	
Dioscorea bulbifera, D. Polystachya, D. cayenensis, D. alata	28°C/5°C 12 h light/12 h dark for 3 weeks	DV	10 or 15% sucrose 3 days-> 13.7% sucrose+18.4% glycerol 20 min → PVS2 20 min	R: 30-47% 3 species; 0% for *D. alata*	[115]
Fragaria × *ananassa* Duch.	4 °C for 2 weeks in the dark	Enc-vit	2 M glycerol + 0.4 M sucrose in beads →PVS2 for 2 h at 0 °C	R: ~90%	[93]
Fragaria × *ananassa* Duch.	5 °C 8 h light/16 h dark for 3 weeks	V-Cryoplate	2 M glycerol + 0.3 M sucrose at 5 °C for 2 days →2 M glycerol + 0.8 M sucrose for 30 min →PVS2 for 50 min	R: average 81% for 15 cultivars	[175]
Fragaria spp. Method tested for 107 cultivars and 20 wild species (51 accessions)	22 °C 8 h light/−1 °C 16 h dark	Vit	5% DMSO and 0.85% agar for 2 days under CA conditions →2.0 M glycerol + 0.5 M sucrose for 15 min →PVS2 for 2.5 h at 0 °C	R: average 89.55% for cultivated accessions and 85.5% for wild accessions	[92]
Juglans regia, 4	22°C/−1°C 8 h	Vit	0.3 M sucrose for 2 days →2 M	R: 59.9-67.8% vs ~0%	[97]

genotypes	light/16 h darkness for 5 weeks, 10 μmol m^{-2} s^{-1}		glycerol + 0.4 M sucrose for 20 min →PVS2 80 min at 0°C	without CA	
Juncus decipiens	5 °C 8 h light/16 h dark for 1-2 months	D-Cryoplate	0.3 M sucrose at 5 °C for 2 days → 2 M glycerol + 1.0 M sucrose 30 min	86.3% average of 20 genotypes.	[97]
Juncus effusus line 'NZ219'	5 °C 8 h light/16 h dark for 30 days	Vit	0.3 M sucrose at 5°C for 3 days →2 M glycerol + 0.4 M sucrose 30 min →PVS2 for 40 min	R: ~70% after 30-60 days of CA vs ~23% without CA	[116]
Lilium spp. Method applied to 160 accessions	4 °C 16 h light/8 h dark, 35 μmol m^{-2} s^{-1} for 7 days	DV	0.3 M sucrose 24 h →0.7 M sucrose 17-24 h →35% PVS3 for 40–60 min → PVS3 for 90-240 min	R: 54.3-58.5% for 160 accessions	[72,117]
Malus domestica, 4 cultivars, *M. sieversii,* 1 cultivar	22°C/−1°C 8 h light/16 h dark for 3 weeks	Vit	0.3 M sucrose for 2 days under CA conditions →PVS2 80 min 0 °C	R: ~60-80% (65% average of 5 cultivars) vs 10-12% average R without CA	[97]
		Enc-Deh	0.75 M sucrose in beads → 0.75 M sucrose for 18 h	R: ~55-80% (65% average of 5 cultivars) vs 5-20% without CA	
Malus spp., 4 cultivars	5°C 8 h light/16 h dark for 3 weeks	CF	5% glycerol+5% DMSO at 0 °C for 30 min	R: 33-78% vs 0-24% without CA	[90]
		Vit	0.7M sucrose for 24 h at 5 °C → PVS3 80 min	R: 53-88% vs 0-22% without CA	
		Enc-Deh	Encapsulated shoot tips precultured with 1.0 M sucrose for 24 h	R: 36-75% vs 0-50% without CA	
Mentha x piperita (3 accessions), *M. x villosa* (3 accessions), *M. spicata* (2 accessions)	25°C/-1°C, 16 h light/8 h dark for 4 weeks or varying durations	DV	0.3 M sucrose 20-24 h →2 M glycerol + 0.4 M sucrose for 2 h → PVS2 for 20 min	R: 57-86% vs 22-53% without CA	[96]
Mentha spicata	4 °C 12 h light/12 h dark for 3 weeks	Enc-vit	2 M glycerol + 0.4 M sucrose for 1 h →PVS2 for 3 h at 0 °C	R: 87% vs max. 63% without CA	[94]
Mentha x piperita genotype 'MEN 198'	25°C/−1°C, 16 h light/8 h dark for 3 weeks	Enc-deh	0.3 M sucrose 24 h →alginate beads with 0.35 M sucrose	R: 62%	[176]
Mentha x piperita nothosubsp. citrate, *M. canadensis*, *M. australis*, and *M. cunninghamii*	22 °C 8 h light / −1°C 16 h dark for 2 weeks	En-deh	0.4 M sucrose in beads →0.75 M sucrose for 18 h	R: ~40-87%	[177]
		Vit	48 h 5% DMSO under CA conditions →2 M glycerol + 0.4 M sucrose →PVS2 for 20 min	R: ~60-86%	[177]
		CF	5% DMSO for 48 h in CA conditions →10% each PEG 8000, glucose, and DMSO	R: ~86-95%	[177]
Pyrus koehnei, P. communis, P. communis x P. pyrifolia, P. pashia, P cordata (8 genotypes)	22°C/−1°C 8 h light/16 h darkness for 1-12 weeks	CF	medium with 0.35% agar/ 0.185 gelrite+5% DMSO for 48 h under CA condition →10% PEG8000+ 10% glucose+10% DMSO 30 min then 30 min at 0 °C	R: ~65-100% vs ~2-16% of non-CA shoot tips R:~17% vs ~2% for 1 genotype, genotype-dependent effect of CA	[91]
Rubus spectabilis, R. idaeus, Rubus spp. 2 accessions	22°C/−1°C 8 h light/16 h dark for 1 week	CF	5% DMSO for 48 h →10% PEG8000+ 10% glucose+10% DMSO for 1 h at −1 °C	S: 51-67% vs 18-41% without CA, genotype-dependent effect of CA, one accession non-responsive	[87]
Rubus spp., 5 genotypes	22°C/−1°C 8 h light/16 h dark + 50 μM ABA for 1 week	CF	5% DMSO for 48 h →10% PEG8000+ 10% glucose+10% DMSO 1 h at 0°C	S: 45.9-84.2% vs 16.9-27.5% without CA, genotype-dependent effect of CA and ABA	[88]
Rubus idaeus, 2	8 °C in darkness for 3	CF	6% sucrose+5% DMSO + 5 mg L^{-1}	R: 45%	[146]

varieties	months, $CaCl_2$ replaced by $Ca(NO_3)_2$, 6% sucrose + 5 mg L^{-1} BA		BA →6% sucrose + 7% DMSO		
	8 °C in darkness for 3 months, $CaCl_2$ replaced by $Ca(NO_3)_2$, 6% glucose + 0.6 mg L^{-1} TDZ	Quick freezing	6% glucose + 5% DMSO + 0.6 mg L^{-1} TDZ →6% glucose + 7% DMSO	R: 70%	[146]
Solanum tuberosum cvs. "Atlantic' and 'Superior'	10 °C for 3 weeks	Vit	0.3 M sucrose 24 h →2 M glycerol + 0.6 M sucrose 40 min →PVS2 at 0 °C for 30 min	R: 51.5% and 11.7% vs <10% without CA	[178]
Solanum commersonii, S. juzepcukii, S. ajanhuiri, S. tuberosum cv. Desiree	6 °C for 2 weeks	DV	2 M glycerol + 0.4 M sucrose 20 min →PVS2 at 0 °C for 50 min	R: 0-57%, genotype-specific response	[102]
Solanum tuberosum spp., S. tuberosum subsp. andigena, S. x juzepczukii, S. x ajanhuiri,S. commersonii (8 genotypes)	6 °C for 3 weeks	DV	2 M glycerol + 0.4 M sucrose 15 min →PVS2 at 0 °C for 50 min	R: average 48% vs 36% without CA. genotype-specific response	[179]
Solanum spp. Method applied to over 4000 accessions	7±2 °C for 2-3 weeks (10-20 μmol m^{-2} s^{-1} for 16-h photoperiod	DV	2 M glycerol + 0.4 M sucrose 20 min →PVS2 at 0 °C for 50 min	R: average 63.5% (20-100% range within species/ subspecies)	[99,100]
Solanum tuberosum 'Desiree', 'King Edward', *S. acaule, S. demissum*	22°C/8°C (8 h light/16 h dark) for 1 week	DMSO droplet	3% sucrose + 0.5 mg L^{-1} zeatin riboside + 0.5 mg L^{-1} IAA + 0.2 mg L^{-1} GA_3 overnight → same solution + 10% DMSO for 2 h	R: average 58.7% (44.6-85.6%) vs 38.4% (19.0-67.4) without CA	[83]
Syringa vulgaris, 2 varieties	8 °C 16 h photoperiod for 2 weeks modified macronutrients+6% sucrose+0.2 mg L^{-1} BA + 1.0 mg L^{-1} PBZ	Prec-deh	273.84 g L^{-1} sucrose + 10 g L^{-1} agar at 0–2 °C for 48 h →air dehydration	R: 62–87%	[118]
Ullucus tuberosus	5°C for 3-4 weeks	D Cryoplate	0.3 M sucrose 16 h →0.4M sucrose in beads →2.0 M glycerol + 1.0 M sucrose 90 min	R: 73-97% (average 90%) for 11 genotypes	[119]
Vaccinium uliqinosum, V. ovatum, V. corymbosum	22°C/−1°C 8 h light/16 h dark for 3, 5, or 7 weeks	CF	Preculture with 5% DMSO for 48 h →10% PEG8000+ 10% glucose + 10% DMSO on ice, then 30 min at −1°C	S: 58% vs 6% without CA for *V. corymbosum,* genotype-dependent effect of CA, some accessions non-responsive	[89]

* Vit, vitrification; DV, droplet-vitrification; Enc-deh, encapsulation-dehydration; Enc-vit, encapsulation-vitrification; Prec-deh, preculture-dehydration; CF, controlled (slow) freezing

** Conditions used to achieve maximum regrowth; DMSO, dimethylsulfoxide; PEG, polyethylene glycol; PVS2: 30% glycerol + 15% DMSO + 15% EG 15.0 + 13.7% sucrose; PVS3: 30% glycerol + 30% sucrose. Treatments were performed at room temperature (23 to 25°C) unless otherwise stated.

*** Best regrowth reported or average regrowth among genotypes
